# P-1172. Staphylococcus Aureus Long-Term Catheter-Related Bloodstream Infection in Children: Clinical Features and Outcome

**DOI:** 10.1093/ofid/ofae631.1358

**Published:** 2025-01-29

**Authors:** Guadalupe Perez, Mariel Merñiez, Emilia Padilla, Moira Taicz, Fernando Berrondo, Ana Paula Arias, Vanesa Reijtman

**Affiliations:** Epidemiology and Infectious Diseases Department. Hospital de Pediatria Garrahan, Ciudad Autonoma de Buenos Aires, Argentina; Juan P Garrahan Hospital, Buenos Aires city, Ciudad Autonoma de Buenos Aires, Argentina; Juan P Garrahan Hospital, Buenos Aires city, Ciudad Autonoma de Buenos Aires, Argentina; Juan P Garrahan Hospital, Buenos Aires city, Ciudad Autonoma de Buenos Aires, Argentina; Juan P Garrahan Hospital, Buenos Aires city, Ciudad Autonoma de Buenos Aires, Argentina; Juan P Garrahan Hospital, Buenos Aires city, Ciudad Autonoma de Buenos Aires, Argentina; Hospital de Pediatria Juan P Garrahan, Capital Federal, Ciudad Autonoma de Buenos Aires, Argentina

## Abstract

**Background:**

*Staphylococcus aureus* long-term catheter-related bloodstream infection (LT-CRBI) are a frequent cause of morbidity and mortality. In *Staphylococcus aureus* LT-CRBI, it is recommended to remove the catheter. However, in pediatrics, management without catheter removal is often necessary.

**Aims:**

to describe the clinical characteristics and outcome of *Staphylococcus aureus* LT- CRBI, to cmpare the clinical features and outcome according to removal or retention of the catheter

**Methods:**

Retrospective cohort study. All patients with *Staphylococcus aureus* LT-CRBI from 1/1/2018 to 12/31/2022 in a pediatric hospital were included. Episodes of relapse of bacteremia were excluded. LT-CRBI was defined according to CDC criteria.

**Results:**

Forty-five patients were included. Median age was 80 months (IQR 27-137). All patients had any underlying disease. Most common comorbidities were onco-hematological disease 27 patients (60%), intestinal failure 5 (11%), chronic kidney disease 3 (7%). Thirty-three were under immunosuppressive treatment (73%).

Median length of bacteremia was 4 days (IQR 3-5) Five patients presented metastatic infection (11%) including 2 patients with pneumonia, 2 thrombophlebitis and 1 soft tissue infection. Twenty-two catheters (49%) were removed. Reasons for removal were: mechanical dysfunction in 6 patients, catheter was not being used in 5 patients, tunnel or pocket infection in 5, septic shock 3 and persistent bacteremia in 3. Two patients died related to infection (4%). *Staphylococcus aureus* methicillin sensitive predominated (78%). Comparing patients with catheter removal vs retained: there were no differences in epidemiological, clinical features and outcome (metastatic infection 13% vs 9%, intensive care admission 3% vs 3%, length of bacteremia 4 days in both groups, mortality 20% vs 17%, respectively)

**Conclusion:**

In this cohort of children with *Staphylococcus aureus* LT- CRBI, catheters were maintained in 51% of patients. No difference in outcome or complications was observed.

**Disclosures:**

**All Authors**: No reported disclosures

